# Hand-Rearing Reduces Fear of Humans in European Starlings, *Sturnus vulgaris*

**DOI:** 10.1371/journal.pone.0017466

**Published:** 2011-02-25

**Authors:** Gesa Feenders, Melissa Bateson

**Affiliations:** Centre for Behaviour and Evolution, Institute of Neuroscience, Newcastle University, Newcastle upon Tyne, United Kingdom; University of Lethbridge, Canada

## Abstract

Pending changes in European legislation ban the use of wild-caught animals in research. This change is partly justified on the assumption that captive-breeding (or hand-rearing) increases welfare of captive animals because these practices result in animals with reduced fear of humans. However, there are few actual data on the long-term behavioural effects of captive-breeding in non-domestic species, and these are urgently needed in order to understand the welfare and scientific consequences of adopting this practice. We compared the response of hand-reared and wild-caught starlings to the presence of a human in the laboratory. During human presence, all birds increased their general locomotor activity but the wild-caught birds moved away from the human and were less active than the hand-reared birds. After the human departed, the wild-caught birds were slower to decrease their activity back towards baseline levels, and showed a dramatic increase in time at the periphery of the cage compared with the hand-reared birds. We interpret these data as showing evidence of a greater fear response in wild-caught birds with initial withdrawal followed by a subsequent rebound of prolonged attempts to escape the cage. We found no effects of environmental enrichment. However, birds in cages on low shelves were less active than birds on upper shelves, and showed a greater increase in the time spent at the periphery of their cages after the human departed, perhaps indicating that the lower cages were more stressful. In demonstrating reduced fear of humans in hand-reared birds, our results support one of the proposed welfare benefits of this practice, but without further data on the possible welfare costs of hand-rearing, it is not yet possible to reach a general conclusion about its net welfare impact. However, our results confirm a clear scientific impact of both hand-rearing and cage position at the behavioural level.

## Introduction

Much laboratory research in animal behaviour involves the use of wild-caught, non-domesticated species. For example, the European starling, which is among the most commonly used passerine species in laboratory research [1], cannot be bred in captivity because the chicks usually die soon after hatching due to a lack of appropriate food. Thus, the birds are generally captured from the wild as juveniles or adults [2]. However, the use of wild-caught animals is likely to become more difficult in the future, because pending changes in European legislation include a ban on the use of wild-caught animals in research [3]. Researchers will instead be required to either captive-breed or alternatively hand-rear wild-caught animals for use in procedures unless strong scientific arguments can be provided demonstrating why this is inappropriate. This change is worrying, given the many lines of evidence that early life experiences, including the developmental environment, maternal deprivation and human handling, can profoundly alter subsequent morphology, physiology and behaviour [4], [5], [6], [7], [8], [9]. Thus, data obtained from captive-reared animals may not be directly comparable with previous data obtained from wild-caught animals. Behavioural research is likely to be particularly adversely affected, since many studies aimed at understanding the proximate and ultimate causes of natural behaviour patterns rely on the assumption that measurements made on animals in the laboratory are indicative of their natural, adaptive responses.

The proposed benefits of captive breeding include the reduced impact of laboratory research on natural animal populations, and also some assumed welfare benefits arising from animals being raised in closer proximity to humans. In both rodents and poultry there is strong evidence for a beneficial role of early human exposure in down-regulating stress reactivity and reducing fearfulness in adults [5], [10], [11]. Since freedom from fear is generally regarded as a cornerstone of good welfare (“Five Freedoms”: [12]), reduced fearfulness would be regarded as a positive welfare outcome of captive breeding.

However, these potential benefits of captive breeding need to be set against the potential costs. In addition to being extremely expensive to implement, captive breeding may also involve some welfare costs, including having to hold animals for much longer in captivity than when using animals caught from the wild at the appropriate age, being unable to release captive-bred animals to the wild at the end of a study, and an increased probability of the development of abnormal behaviour patterns such as stereotypies [13], [14]. Captive-breeding of wild animals is often associated with hand-rearing and consequent maternal deprivation, which is associated with altered behaviour and poor welfare outcomes [13]. In direct contrast to early handling, maternal deprivation in rats has been shown to be associated with the development of high stress reactivity and generally increased fearfulness (e.g. [15]). Therefore, in deciding whether and under what conditions captive breeding and/or hand-rearing is appropriate, a careful cost-benefit analysis is essential. However, a major problem we face is the lack of good scientific data on the above issues, especially from the non-domesticated animals regularly used in ethological research such as passerine species including starlings and corvids [1]. This lack of knowledge was recognised in the report of the Group of Experts on birds for the Council of Europe Convention ETS123, which states that “more research is needed into the effects of handling chicks from hatch on subsequent handling stress in adult birds.” [16].

With a focus on non-domesticated avian species, there are only few studies, primarily on parrots, exploring the effect of early handling on subsequent behaviour. A survey on African grey parrots (*Psittacus erithacus*) showed that chicks taken from the nest prior to 5 weeks of age had a greater probability of subsequently developing stereotypies, and invasive rearing methods (e.g. tube-feeding) resulted in birds that were more aggressive towards humans [17]. In contrast, in orange-winged Amazon parrots (*Amazona amazonica*) early handling reduced the chicks’ fear of humans and stress reaction to restraint combined with an increased immune response [18], [19]. Our own observations of hand-reared starlings showed that the birds’ tameness disappeared as soon as the birds became fully independent leading us to question whether there were any lasting effects on fearfulness. Thus, given the equivocal nature of the limited evidence currently available, we urgently need more data on the wild bird species typically used in behavioural studies to establish whether there are indeed benefits to hand-rearing.

The aim of the current study was to explore the long-term effects of hand-rearing on the behaviour of the European starling (*Sturnus vulgaris*) [1]. More specifically, we set out to ask whether hand-reared starlings taken from the wild as young nestlings are less fearful of humans than birds caught from the wild after reaching independence. One of the standard procedure for testing fear of humans in fowl is the open field test (e.g. [20], [21]). However, we did not want to subject the birds to a novel and potentially stressful test arena, so instead we left the birds in their home cages and measured their response to a human entering and standing in the room containing the cages (more comparable to a newer test procedure as used in [22]). We measured the birds’ general activity and their use of different cage locations before, during, and after the human entered the room.

There is some evidence that the current cage environment can affect the fearfulness of caged birds. Starlings housed in cages enriched with branches, water baths and a substrate for probing have been found to demonstrate more optimistic behaviour, possibly indicative of a more positive affective state characteristic of reduced anxiety [23], [24]. Therefore, in order to study any interactions between rearing and current environment on fearfulness, we used a two-way factorial design in which wild-caught and hand-reared birds were housed in either non-enriched or enriched cages.

We predicted that the hand-reared birds, if they exhibited reduced fear of humans compared to the wild-caught birds, would show more time spent in the front part of the cage nearest to the human during the period of human presence, and fewer escape attempts indicated by a reduced use of peripheral cage locations such as the top corners [25]. On the basis of our previous findings from starlings we predicted that environmental enrichment would also reduce fear.

## Methods

### Ethics statement

Our study adhered to the Association for the Study of Animal Behaviour’s Guidelines for the Use of Animals in Research and also passed the Newcastle University Ethical Review Committee. The starlings were taken from the wild under Natural England licence number 20093194. Birds were released back into the aviaries after the experiment and retained for further studies.

### Animals

We used a total of 16 hand-reared (7 males, 9 females) and 16 wild-caught (7 males, 9 females) European starlings *(Sturnus vulgaris)*, but one wild-caught female died after having been in the cage for 7 days. The hand-reared birds were taken in May 2009 at 6–12 days post-hatching from nest boxes located on farm buildings around Northumberland. Only one chick was taken from each box to avoid pseudoreplication. The birds were transferred to the laboratory for hand-rearing. They were housed in artificial nests lined with tissue paper and covered loosely between feeds. They were fed a mix of soaked dry cat food and apple sauce, supplemented with vitamins (BSP drops, Vetark) and calcium (Zolcal D, Vetark). Initially the chicks were fed approximately every 30 minutes for 14 hours per day, but the frequency of feeds was gradually reduced as the birds grew. At around 3 weeks of age when the chicks fledged and started to feed themselves they were transferred to a large indoor aviary (3.60×2.40×2.25 m WDH) enriched with bark chips (natural probing substrate) and a water bath.

The wild-caught birds were caught with a baited whoosh net as juveniles at the end of September 2009 from the same population as the hand-reared birds. They were immediately transferred to an indoor aviary similar to that used for the hand-reared birds so that hand-reared and wild-caught birds were always kept in separate aviaries and had no contact with each other. All birds in the aviaries were provided with ad libitum food (chick crumbs) and water, supplemented with dried insect food (Insect Patee, Orlux), fruit and mealworms. The temperature in the aviaries was kept between 17–19°C, with a constant light period of 14L:10D. The rationale for keeping both groups fully separate was to replicate the conditions that would be present in a research lab following the revised EU directive, i.e. to rely exclusively on hand-reared starlings. In contrast, if we had housed the birds in mixed groups, they could have influenced each other’s behaviour; for example, the wild-caught birds might be more frightened by the human care-taker and induce more escape responses in hand-reared birds, or, alternatively, the hand-reared birds might be less scared of human care-takers and increase habituation in the wild-caught birds. Such influences would have reduced the validity of our comparison.

The experiment described in the current paper commenced in November 2009 when all birds were approximately 6 months of age; the short breeding season of starlings in the North-East of England only allows for one brood, meaning that all our birds were of very similar age when tested. Prior to testing, the wild-caught birds had been in captivity for a minimum of 4 weeks (required quarantine period with anti-parasite treatment every 10 days).

### Experimental set up

**Figure 1:**
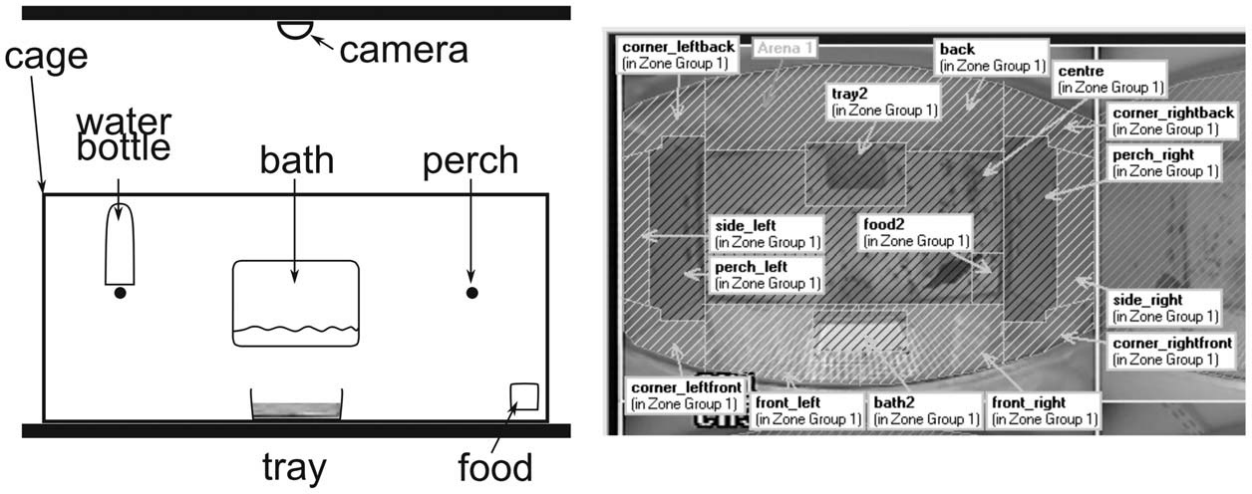
Experimental set up. Left panel: side view of a cage with furnishings. Right panel: top view of a cage as seen on the video images. For the automatic tracking of the bird in the cage, distinct locations of the cage were allocated to areas of this image. Areas hatched in white were combined as “peripheral locations” for analysis (this must not be confused with the “front” and “back” section (not illustrated), where the full cage is split along its longitudinal axis).

For the experiment, starlings were individually housed in cages (100×45×45 cm WDH) constructed with solid floors and side walls, wire mesh fronts and backs and transparent Plexiglas roofs. Eight such cages were arranged on two rows of shelves (at 38 and 120 cm height, designated ‘low’ and ‘high’ respectively) in the experimental room such that there were four cages on the low level and four on the high level; the arrangement allowed each bird to see 4 to 6 other birds. Each cage was fitted with an overhead surveillance camera (Atom, CSP Technology, UK) connected to a computer in a separate room that could be used for remote observation and video recording. Four of the cages (two high and two low) were environmentally enriched with a plastic tray filled with wood chips as a natural probing substrate, a water bath filled with water at all times, and a little hide on the distal end of one of the two perches (Figure 1a). Previous studies have shown that these enrichments are likely to improve the welfare of caged starlings [23], [24], [26]. The other four cages were fitted with empty plastic tray and water bath. In the non-enriched cages the bath was filled twice a week for one hour to ensure good hygiene.

Since the experimental room only housed eight birds, the study comprised four replicate groups, each consisting of four hand-reared and four wild-caught starlings assigned to the cages in a pseudo-randomized and fully counterbalanced way. Each group was kept in the individual cages for 14 days before being returned to their aviaries and the next group being moved in. Each group started on a Monday (day 0) to keep possible effects of weekends constant.

### Testing the response to human presence

We tested the birds’ response to a human on the afternoon of day 9 during a period when the birds were mostly resting and feeding. The experimenter (GF) entered the experimental room dressed in the normal protective clothing worn during daily husbandry (white lab coat, green hat, face mask). Standing in the centre of the room, the experimenter then faced one column of two cages for 30 sec before slowly rotating ∼90° on the spot to face the neighbouring column of cages, continuing like this until two full rotations were completed before finally leaving the room. Throughout the human-presence period, which lasted 255 sec, the experimenter directed her eyes towards a timer held in both hands to prevent her direct gaze being an additional stressor [27]. Due to the cage arrangement in the room, the distance between the experimenter and each individual cage was different (approximately 80–130 cm to the nearest cage corner) and the viewing angle differed within and between cages (roughly, when sitting at the back of their cages, birds in the high cages would not see the lower legs and feet of the experimenter, whereas birds in the low cages would not see the shoulders and head; all birds could see the full body-length of the experimenter at least when close to the front-wall of the cage); however, as we used a counterbalanced design with respect to origin of bird and housing, across the four replicate groups, this variation should not bias the results.

The experimenter was a person familiar to the hand-reared birds as she had been the main person involved in the actual hand-rearing protocol. All birds had repeated contact with the experimenter during the days kept in the cages prior to this test as other tests were conducted requiring the experimenter to enter the room for a few minutes each day (Feenders et al., submitted).

### Data collection

We recorded the birds’ behaviour using the video cameras from 10 minutes prior to the experimenter entering the room until 15 minutes after the experimenter had left the room. We divided the videos into 5 periods of 255 seconds (the duration of the human presence): pre-1, pre-2, presence, post-1, post-2 and post-3. The videos were automatically analysed using the tracking software EthoVision XT v5.1 (Noldus Information Technology, Wageningen, Netherlands). This software is based on a contrast-detection algorithm to detect target objects by comparing images, and the dark starling on the light cage background (white paper was used to line the floor of the cages) was reliably detected and tracked. We used a sample rate of 2.5 frames per second for analysis optimised beforehand for accuracy (by comparison with manual scoring) and time efficiency. 

We divided the top view image of the cage into areas that corresponded to distinct cage locations sufficiently detailed to record the bird’s movements, e.g. “left perch”, “tray”, “water bath”, “corner at left front top” (Figure 1b). In addition, we divided the top view of the cage into a front (nearest the human) and a back half to investigate the response of the birds to the human; note that this latter classification is independent of the above mentioned cage locations.

We used Ethovision to extract the following three behavioural variables: T(move) which was equal to the total length of time the bird spent moving (>10 cm/s); T(front) which was equal to the total time the bird spent in the front half of the cage; and T(peripheral) which was equal to the total time spent in peripheral locations, i.e. clinging to the cage walls and the top corners of the cage (Figure 1b).

### Statistical analysis

All statistical tests were done using SPSS 17.0. To explore the effects of developmental origin and current housing on the reaction of the birds to the presence of the experimenter we used general linear models (GLMs) to model the data. In the first series of GLMs we focused on just the period of human presence. We used T(move), T(front) and T(peripheral) as our dependent variables. Since these data were bounded between 0 and 255, in order to obtain normally distributed data we expressed the times as a proportion of the maximum and then applied the arcsine square-root transformation. Independent variables included in the GLM were the three between-subjects factors: origin of birds (origin: hand-reared versus wild-caught), housing condition (housing: enriched versus non-enriched), and replicate group (group: 1–4).

In a second series of GLMs we focused on the change in the birds’ behaviour when the experimenter left the room. We used as our dependent variables the difference in behaviour (for each of the three measures described above) between the period of human presence and the period immediately after the experimenter had left the room (presence - post-1); these difference measures were not transformed. As before, the independent variables included were the between-subjects factors origin, housing, and group.

In both series of analyses we included the interaction between origin and housing in our models but no interactions with replicate group, because replicate group was deemed to be an arbitrarily assigned blocking factor unlikely to have a non-additive interaction with our main treatments [28]. In cases where our data did not meet the assumption of homogeneity of variance (Levene’s test p < 0.05), and further transformations did not correct the problem, we resorted to a non-parametric two-way Kruskal-Wallis test [29]; by necessity, group was omitted from these non-parametric analyses. Non-parametric analyses are indicated in Table 1.

**Table 1:** Statistics from the time during the human presence and in comparison with the post-1 period.

	presence			presence – (post-1)		
	T(move)	T(front)	T(peri-pheral)	T(move)	T(front)	T(peri-pheral)
**Model: origin, housing, origin x housing**						
origin	2.63, >0.100 †	**14.05, 0.001**	0.18, 0.676	**6.08, 0.021**	**10.67, 0.003**	3.42, 0.077
housing	0.51, >0.250 †	0.76, 0.393	0.02, 0.897	0.02, 0.903	2.39, 0.136	0.76, 0.391
origin x housing	0.69, >0.750†	0.47, 0.500	0.12, 0.737	0.01, 0.942	0.24, 0.626	1.43, 0.243
**Model: origin, cageposition, origin x cageposition**						
origin	**6.60, 0.017**	**13.34, 0.001**	0.14, 0.715	**8.08, 0.009**	**7.78, <0.010** †	3.33, 0.080
cagepos.	**7.75, 0.010**	0.02, 0.898	0.11, 0.747	**10.49, 0.003**	0.57, >0.250 †	**6.09, 0.021**
origin x cagepos.	0.73, 0.402	0.00, 0.996	0.61, 0.441	1.74, 0.199	0.05, >0.750 †	2.69, 0.114

The top half of the table shows the results from the first set of models with origin and current housing; the bottom half of the table shows the results from the second set of models with origin and cage position. Each cell contains the relevant *F*-ratio (*df*  =  1,24 in all cases) followed by the associated *P*-value.

† Kruskal-Wallis test (shown are *H*_1_- and *P*-values). Significant effects (*P* < 0.05) are highlighted in bold.

Third, we examined the change over time in the behaviour of the birds during the three post-presence time periods. We performed a repeated-measures GLM with period (post-1, post-2 and post-3) as the within-subjects factor, and the same between-subject factors used above. Arcsine square root transformed proportions of T(move), T(front) and T(peripheral) were used as the dependent variables in these analyses. For all repeated measures GLMs, the reported p-values associated with the F-ratios are adjusted using the Greenhouse-Geisser correction to deal with cases in which sphericity was violated (Mauchly’s sphericity test p < 0.05).

Since we did not find any effect of the environmental enrichment provided but noticed some prominent differences between the high and low cages, we performed a second series of analyses, similar to those described above, but now with position of the cage (cageposition: low versus high), origin, and group as between subject factors. Note that cage position was not included in the original analyses because the experiment was designed such that it was completely counterbalanced, and thus could not confound the results. The same is true of current housing for the latter analyses.

## Results

**Figure 2:**
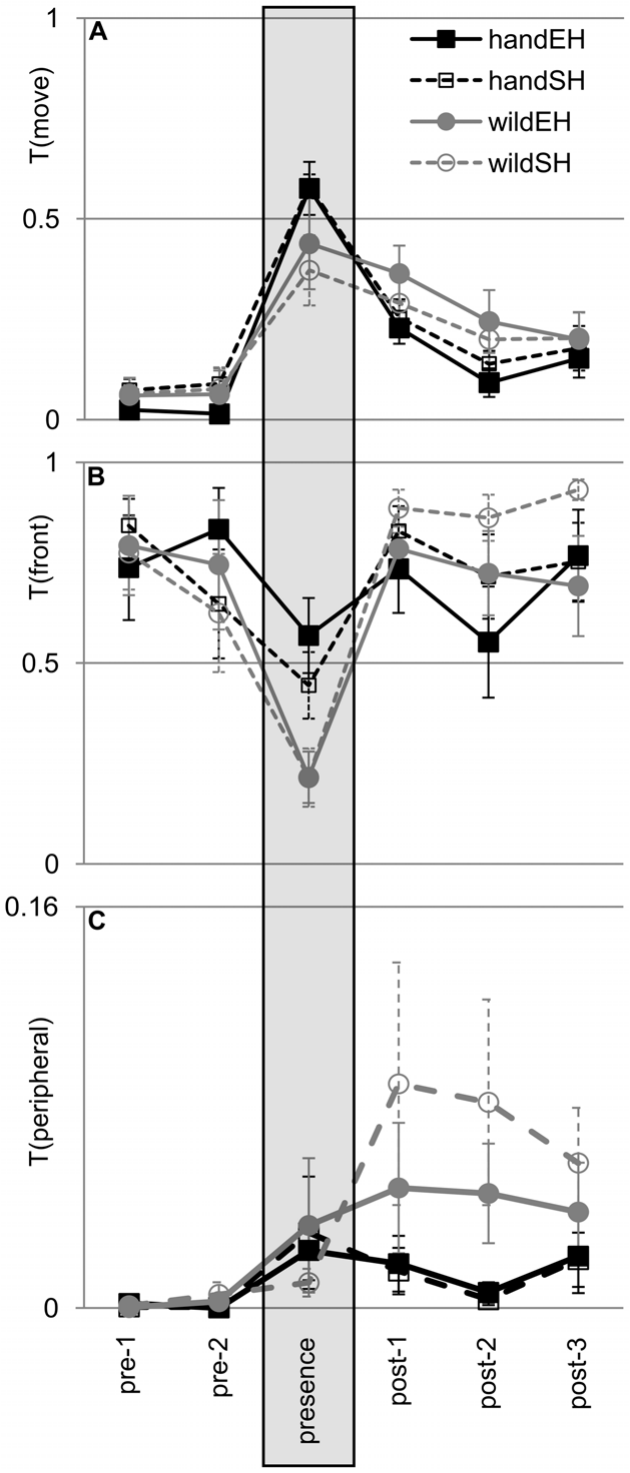
Response to a human. Effect of origin and housing on (A) general activity T(move), (B) use of front section of cage T(front), and (C) use of peripheral cage locations T(peripheral). Shown is the behaviour over the course of 6 consecutive time periods of 255 sec duration each. The grey shaded box indicates the period when the human was present. Pre-1 and pre-2: periods before the human entered the room; post-1, post-2 and post-3: periods after the human had left. Black squares: hand-reared birds; grey circles: wild-caught birds; filled symbols, solid lines: enriched housing (EH); open symbols, dashed lines: non-enriched housing (SH). Shown data values are not transformed but normalized to the length of the time period (thus, a value of 1(100%) is equivalent to 255 sec, and a value of 0.5 (50%) to 127.5 sec). Data show group means ±1 SEM.

Figure 2 summarizes the results for the three behavioural variables we explored. General activity (T(move)) was low in the periods prior to the experimenter entering the room (pre-1 and pre-2), increased dramatically during human presence and then declined again after the experimenter had left the room (post1, post-2 and post-3), although not to the pre-entry levels within the 15 minutes for which data were collected (Figure 2a). Time spent in the front of the cage nearest to the where the experimenter stood (T(front)) was high prior to the experimenter entering the room, reduced dramatically during human presence and then increased back to pre-entry levels again after the experimenter had left the room (Figure 2b). Time spent in peripheral locations (T(peripheral)) was negligible in the periods prior to the experimenter entering the room, increased during human presence and then showed a treatment-dependent change after the experimenter had left the room (Figure 2c). For all three behavioural variables, treatment effects are seen in the responses of the birds during and/or after human presence; this variability is explored in detail in the following analyses.

### Effect of origin and housing

**Figure 3:**
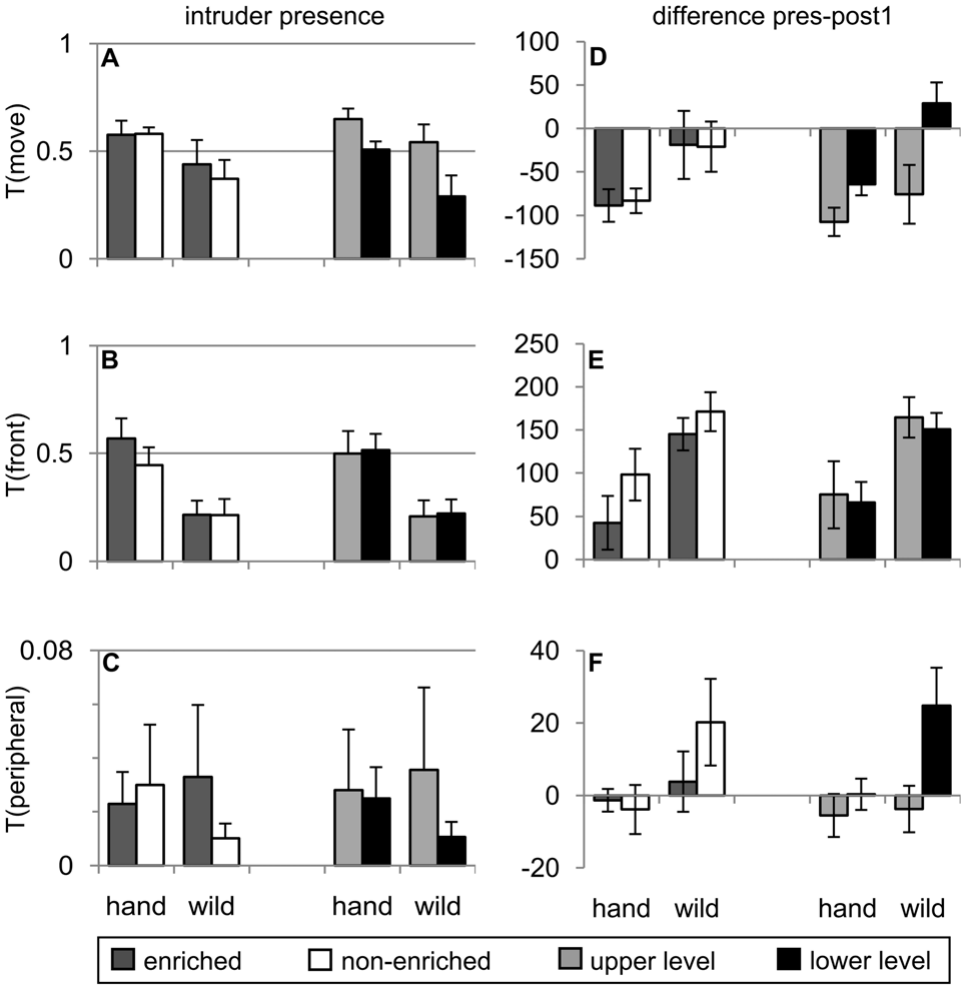
Effects of cage enrichment and position. Starlings' reaction to human presence (A,B,C) and change in behaviour upon departure of the human (D,E,F). Shown are the effects of environmental enrichment (left four bars; dark grey: enriched, white: non-enriched) and cage position (right four bars; light grey: high cages, black: low cages) in combination with origin (hand: hand-reared birds; wild: wild-caught birds). Shown data values in A, B, C are normalized to the length of the time period. Data show group means ±1 SEM.

During the human-presence period, origin had a significant effect on T(front) with the wild-caught birds spending less time in the front section (nearer to the experimenter) than the hand-reared birds. We found no effect of origin on either T(move) or T(peripheral). We found no effect of housing, or origin x housing interaction on any of the three measures. Statistics (F-ratios and p-values) for the above analyses are summarised in Table 1 and means are displayed in Figure 3a–c.

The change in behaviour from the human-presence period to the consecutive period (post-1), was significantly affected by origin for T(move) and T(front), with wild-caught birds showing a smaller reduction in activity and a greater increase in the time spent in the front of the cage than the hand-reared birds (this increase is a result of reduced use of the front section during the human presence and not due to an increase after the human departed). There was no effect of housing, or origin x housing interaction on any of the behavioural measures (Table 1, Fig. 3d–f).

Across the post-presence periods (post-1, post-2 and post-3), T(move) decreased significantly, but T(peripheral) and T(front) did not change. In addition, T(peripheral) was influenced by origin, with wild-caught birds spending more time in peripheral locations. All other effects were non-significant (Table 2).

**Table 2:** Statistics from the time after the experimenter had left the room.

	T(move)	T(front)	T(peri-pheral)
**Model: origin, housing, origin x housing**			
origin	2.32, 0.141	1.38, 0.251	**7.81, 0.010**
housing	0.01, 0.913	3.59, 0.070	0.51, 0.483
origin x housing	0.45, 0.510	0.24, 0.626	0.51, 0.482
time	**48.41, <0.001**	1.65, 0.210	1.17, 0.316
**Model: origin, cageposition, origin x cageposition**			
origin	2.50, 0.127	1.06, 0.313	**8.29, 0.008**
cagepos.	3.62, 0.069	0.46, 0.504	**5.41, 0.029**
origin x cagepos.	2.75, 0.110	0.58, 0.456	0.39, 0.539
time	**47.03, <0.001**	1.66, 0.206	1.21, 0.305

Results from repeated measures GLM. The top half of the table shows the results from the first set of models with origin and current housing; the bottom half of the table shows the results from the second set of models with origin and cage position. Each cell contains the relevant *F*-ratios and *P*-values (origin, housing/cageposition, origin x housing/cageposition *df*  =  1,24; time *df*  =  2,48). Significant effects are highlighted in bold.

### Effect of origin and cage height

During the human-presence period origin had a significant effect on T(move) and T(front), with the wild-caught birds spending less time moving and in the front section of the cage; T(peripheral) was unaffected. Cage position had an additional significant effect on T(move) with birds in the low cages spending less time moving, but no effect on T(peripheral) or T(front). There was no effect of the origin x cageposition interaction (Table 1, Fig. 3a–c).

The change in behaviour from the human-presence period to the consecutive period, was significantly affected by origin for T(move) and T(front) as above; cage height also significantly affected T(move) and T(peripheral), but not T(front); there was no effect of origin x cageposition interaction (Table 1, Fig. 3d–f).

Across the post-presence periods (post-1, post-2 and post-3), T(move) decreased, but T(peripheral) and T(front) did not change. In addition, T(peripheral) was influenced by origin and cage height, with both the wild-caught birds and the birds in low cages spending more time in peripheral cage locations. All other effects were non-significant (Table 2).

Replicate groups did not differ significantly during the intruder-presence period or in their change in behaviour from presence to post-1 (p > 0.1; Supporting Figure S1).

## Discussion

The aim of this study was to explore whether hand-reared European starlings and similar-aged birds caught from the wild as juveniles differ in their fear of humans. We found clear differences in the way the hand-reared and wild-caught birds responded to a human. While the human was present, the birds instantly increased their general activity but the wild-caught birds moved away from the front half of the cage nearest to the human and were less active than the hand-reared birds. After the human had left the room, the wild-caught birds were slower to decrease their activity back towards baseline levels than the hand-reared birds, and showed a dramatic increase in the time spent at the periphery of the cage compared with the hand-reared birds. We found no effects of whether the birds were housed in enriched or non-enriched cages. However, we did find some effects of whether a bird’s cage was located on the low or high shelves in the laboratory. Birds on the lower level were less active than birds on the higher level (independent of their developmental origins), and showed a greater increase in the time they spent at the periphery of their cages after the human had left the room.

We interpret the above behavioural observations as indicative of a greater fear response to the human in the wild-caught birds and also in the birds housed in the lower cages. The movement away from the human during the period of human presence observed in the wild-caught birds and reduced activity are similar to results from chickens. When tested in an open field arena, chickens responded to humans as potential predators, showing freezing and flight behaviour [21]. Although we recorded a reduction in activity levels in the wild-caught birds during the period of human presence, interestingly we did not observe any clear freezing behaviour, but only very short periods (a few seconds) of motionless, perhaps because the birds had habituated to humans entering the room/aviary during daily exposure. The use of peripheral cage locations such as top corners and cage walls has been suggested to be indicative of escape attempts in caged starlings [25]. Thus, it seems that the wild-caught birds exhibited stronger flight behaviour after the human had left as shown by the increased use of peripheral locations compared to the hand-reared birds. We suggest that during the presence of a human, where both groups showed similar use of peripheral locations, the wild-caught birds were more stressed, but because their motivation to escape was thwarted by the human blocking the escape route, especially for the lower cages (see below), their escape attempts were partly suppressed. This motivation was subsequently expressed in the increased and prolonged use of peripheral locations after the human had departed. In contrast, the hand-reared starlings, while clearly reacting to a present human with increased general activity and use of peripheral cage locations, immediately decreased their response when the danger was gone.

The difference in the dynamics of the response to the human in the hand-reared birds mirrors previous findings in rodents showing reduced responses to various stressors with a faster return to baseline levels in early-handled animals [4], [6], [7]. In contrast, the wild-caught starlings showed a delayed, but much greater and more prolonged response to the human stressor that had failed to return to baseline levels by the end of the observation period. Although we have not directly tested the possibility in this paper, it is likely that the observed difference in the response of the wild-caught and hand-reared birds to humans is closely connected to or even driven by a change in general stress reactivity (as above). We are currently collecting data on the physiological stress responses of the starlings to further elucidate this connection.

The experience of the birds during our hand-rearing procedure we employed would have differed from that of the wild-caught birds in several respects including the amount of early human handling received but also the quantity and quality of social interaction with conspecifics. Although during hand-rearing the chicks were kept in small groups in an attempt to mimic natural conditions, the social interaction of parent birds and chicks was totally absent. In addition, chicks in this study were briefly isolated from their nest-mates on a daily basis in order to take their body-weights and to clean the nest boxes. In a previous study, Hausberger et al. [30] showed that the social experience during the first year shaped the response of hand-reared starlings to a human in that birds housed in social groups spent more time close to the human than birds housed singly or in pairs. In our study, the starlings were kept as a group throughout the study, except when being tested in individual cages. Thus, we argue that the behaviour reported in the current study is most comparable to the socially-housed group of Hausberger’s study, and this seems in line with the marked difference of the use of the cage front during the human presence.

An unanticipated finding from our study was the effect of the cage height on the responses of the birds: birds in the lower cages showed a delayed recovery with respect to use of peripheral locations, whereas birds in the upper cages had a direct response to the human but immediately reduced their activity to baseline levels once the human had left the room. Similar to our above argument, it is possible that birds in the lower cages were subjected to more stress because a human standing in the room (during husbandry or testing) was almost fully blocking the potential escape route for the birds. We argue that specifically the wild-caught birds in lower cages were thwarted by the human standing in front of the cage. This then led to a delayed response as soon as the human left, resulting in high levels of movement and increased use of peripheral locations, whereas the birds in the upper cages could perform their escape attempts even during the human presence period and were then settling down once the human disappeared. If birds in the lower cages were more affected by the human (e.g. experiencing higher levels of fear of human because of unusual perspectives) we would expect a higher avoidance of the front section in birds in the lower cages compared to the upper cages during the human presence period (similar to the wild-caught birds), but our results do not support this. An alternative explanation is that birds in the lower cages were exposed to social stress: because the cages of 2–3 other conspecifics housed in the same room were well visible on higher levels, birds in the lower cages may feel subordinate, as the dominant birds usually occupy the most favoured places which are high up [31]. This or other effects of the cage height (e.g. lower light levels) could induce chronic stress and possibly a depression-like state in birds in the lower cages. It is known that animals, including humans, show a delayed recovery from stress response when depressed [32], [33], [34], [35]. Although the development of chronic stress in low-level cages seems likely to us, at this point we do not know whether this is the case in our birds at this early stage of stress exposure; other studies have used more than 14 days of repeated stressor exposure to measure chronic stress reaction [36], [37].

Only very few studies have so far evaluated the potential impact of the cage height/position on the animal’s state (behaviour, welfare, physiology etc). Ader et al [38] showed that mice housed higher on a rack had a delayed onset of diabetes, probably mediated by the emotional state of the animal. Garner and colleagues [39] also showed a negative relationship between cage distance to the room door and abnormal feather picking in parrots. Our results support the conclusion of these studies that, depending on the species and behavioural/physiological measure under observation, cage position has the potential to significantly alter the animal’s behaviour/physiology and should be taken into account when performing animal research.

Interestingly, we did not find any effect of our environmental enrichment manipulations on the behaviour of the birds, showing that an unfavourable cage position can have a stronger impact on a bird’s welfare than the addition of some mild environmental enrichment. Nevertheless, there seems to be a tendency for the wild-caught birds to be more responsive to environmental enrichment: Figure 2c suggests that the wild-caught birds in non-enriched cages show an exaggerated response pattern in their use of peripheral locations. It is possible that a bigger difference in environmental enrichment would have led to more prominent effects.

The observed differences between the hand-reared and wild-caught starlings described in this paper were recorded in birds of between 5 and 8 months of age, at which point the wild-caught birds had been in captivity for between 1 and 4 months. Although this represents a limited time window, and it could be argued that different responses might have been observed had we kept the birds for longer, we argue that the time window we chose offers the best validity for informing researchers’ decisions about the source of birds. Our comparisons took place during the period when behavioural studies would commonly take place, i.e. immediately after completion of quarantine (where required). If better habituation only occurred after longer periods in captivity, the major welfare and financial advantages of using wild-caught birds would be reduced (i.e. the potential for a minimum period in captivity followed by rapid release to the wild following completion of testing). Furthermore, while our first replicate group was tested after the wild-caught birds had been in captivity for 1 month, the last replicate group was tested almost 3 months later yet we did not observe any tendency of replicate group(s) tested later to show less fear response. Thus, we have no evidence to suggest that the wild-caught birds were altering their responses to humans over a period of 4 months in captivity. Therefore, we conclude that the time window chosen for our comparisons seems suitable to draw more general conclusions especially in the light of common laboratory practice.

In conclusion, we have confirmed one of the proposed welfare benefits of captive breeding/hand-rearing in starlings; hand-reared starlings show behaviour that we interpret as evidence of reduced fear of humans compared to wild-caught birds. Additionally, we have shown that starlings respond differently to a human according to their cage position in the laboratory: birds on the lower shelves show evidence of greater stress. This study therefore has direct implications for any researcher planning laboratory-based projects on non-domesticated (bird) species, because birds’ behaviour is likely to be affected by their developmental history and cage position. However, on the basis of the findings presented here we are unable to make a statement about the overall animal welfare benefits of hand-rearing, because the current study has not addressed the potential costs of this practice. For example, it remains to be demonstrated whether hand-reared starlings are more likely to develop abnormal behaviour patterns, such as locomotor stereotypies, as has been reported in other species.

## Supporting Information

Figure S1 **Effect of replicate group.** Effect of origin (different symbols; hand: hand-reared; wild: wild-caught) and replicate group (different colours; numbers indicate replicate groups 1 to 4) on (A) general activity T(move), (B) use of front section of cage T(front), and (C) use of peripheral cage locations T(peripheral). Shown data values are normalized to the length of the time period. Data show group means ±1 SEM.
(PDF)
