# Supplementary material for: Hand-Rearing Reduces Fear of Humans in European Starlings, Sturnus vulgaris
Source: PLoS One. 2011 Feb 25;6(2):e17466. doi: 10.1371/journal.pone.0017466 (PMC3045461; doi:10.1371/journal.pone.0017466)
Supplement: Figure S1 — Effect of replicate group. Effect of origin (different symbols; hand: hand-reared; wild: wild-caught) and replicate group (different colours; numbers indicate replicate groups 1 to 4) on (A) general activity T(move), (B) use of front section of cage T(front), and (C) use of peripheral cage locations T(peripheral). Shown data values are normalized to the length of the time period. Data show group means ±1 SEM. (PDF) [file pone.0017466.s001.pdf]

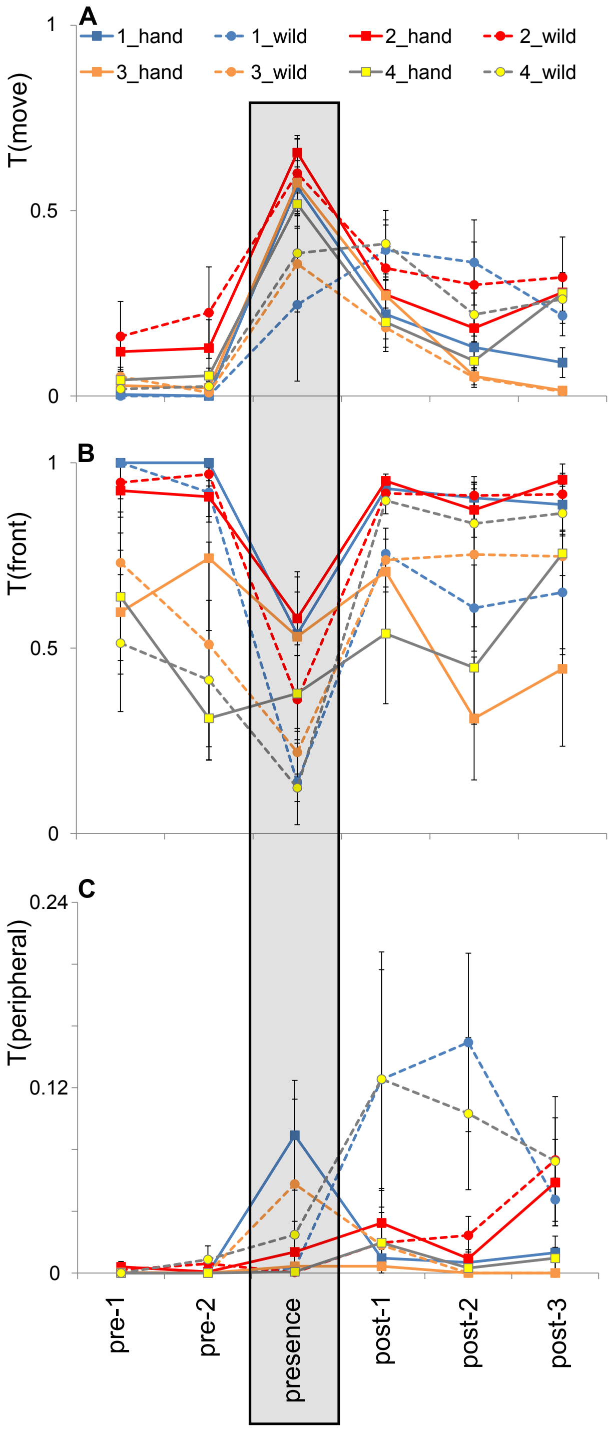

Supporting Figure S1. Effect of replicate group. Effect of origin (different symbols; hand: hand-reared; wild: wild-caught) and replicate group (different colours; numbers indicate replicate groups 1 to 4) on (A) general activity T(move), (B) use of front section of cage T(front), and (C) use of peripheral cage locations T(peripheral). Shown data values are normalized to the length of the time period. Data show group means ± 1 SEM.
